# Embelin, a Potent Molecule for Alzheimer's Disease: A Proof of Concept From Blood-Brain Barrier Permeability, Acetylcholinesterase Inhibition and Molecular Docking Studies

**DOI:** 10.3389/fnins.2019.00495

**Published:** 2019-05-16

**Authors:** Saatheeyavaane Bhuvanendran, Nur Aziah Hanapi, Nafees Ahemad, Iekhsan Othman, Siti Rafidah Yusof, Mohd Farooq Shaikh

**Affiliations:** ^1^Neuropharmacology Research Laboratory, Jeffrey Cheah School of Medicine and Health Sciences, Monash University Malaysia, Bandar Sunway, Malaysia; ^2^Centre for Drug Research, Universiti Sains Malaysia, Penang, Malaysia; ^3^School of Pharmacy, Monash University Malaysia, Bandar Sunway, Malaysia; ^4^Tropical Medicine and Biology Multidisciplinary Platform, Monash University Malaysia, Bandar Sunway, Malaysia

**Keywords:** embelin, blood-brain barrier, permeability, acetylcholinesterase inhibitor, molecular docking, amyloid beta peptides

## Abstract

Embelin is well-known in ethnomedicine and reported to have central nervous system activities. However, there is no report on blood-brain barrier (BBB) permeability of embelin. Here the BBB permeability of embelin was evaluated using *in vitro* primary porcine brain endothelial cell (PBEC) model of the BBB. Embelin was also evaluated for acetylcholinesterase (AChE) inhibitory activity and docking prediction for interaction with AChE and amyloid beta (Aβ) binding sites. Embelin was found to be non-toxic to the PBECs and did not disturb the PBEC barrier function. The PBECs showed restrictive tight junctions with average transendothelial electrical resistance of 365.37 ± 113.00 Ω.cm^2^, for monolayers used for permeability assays. Permeability assays were conducted from apical-to-basolateral direction (blood-to-brain side). Embelin showed apparent permeability (*P*_app_) value of 35.46 ± 20.33 × 10^−6^ cm/s with 85.53% recovery. *In vitro* AChE inhibitory assay demonstrated that embelin could inhibit the enzyme. Molecular docking study showed that embelin binds well to active site of AChE with CDOCKER interaction energy of −65.75 kcal/mol which correlates with the *in vitro* results. Docking of embelin with Aβ peptides also revealed the promising binding with low CDOCKER interaction energy. Thus, findings from this study indicate that embelin could be a suitable molecule to be further developed as therapeutic molecule to treat neurological disorders particularly Alzheimer's disease.

## Introduction

The blood brain barrier (BBB) is highly selective interface that separates the central nervous system (CNS) from the bloodstream (Clark, 2003; Abbott et al., 2010). The BBB is composed of brain endothelial cells that formed the cerebral microvasculature which is interconnected by tight junctions (Abbott et al., 2010; Daneman and Prat, 2015). The endothelium facilitates and regulates substance entry between the blood and the CNS, as well as protecting the brain from harmful toxins and pathogens. Unfortunately, the protective nature of the BBB becomes a disadvantage as it also restricts the entry of many potential therapeutic agents (Czupalla et al., 2014). Newly developed drugs targeting CNS disorders have the poorest success rate and often failed in the clinical trial (Fernández-Ruiz, 2018). Around 98% of the potential drugs do not cross the BBB. Due to their inability or poor ability to cross the BBB, they cannot be utilized for CNS related disorders (Pardridge, 2001) and this imposed major hurdles in pharmacological treatment of CNS disorders (Pathan et al., 2009). Therefore, it is very crucial to know whether a compound can cross the BBB and utilize this information during drug development before proceeding to clinical trial.

*In vivo* BBB methods provide the most reliable measurement for drug permeation due to the complex nature of the BBB, but with limitations of a low throughput and being labor intensive (Abbott, 2004; Patabendige et al., 2013a). Thus, good *in vitro* BBB model which demonstrates restrictive tight junctions reflected by high transendothelial electrical resistance (TEER) (Liew et al., 2017) and resembles the *in vivo* conditions is very important for effective screening for BBB permeability in drug discoveries (Patabendige et al., 2013a; Yusof et al., 2014). Several studies have reported on *in vitro* BBB models from variety of species including from mice, rats, cows, pigs, and human (Franke et al., 2000; Xue et al., 2013; Yusof et al., 2014; Thomsen et al., 2015). However, some of the reported BBB models suffered from low TEER indicating leaky tight junctions (Yusof et al., 2014). For instance, the human cerebral microvascular endothelial cell line (hCMEC/D3) which showed TEER values of <50 Ω.cm^2^ is probably not suitable for BBB permeability studies of small molecules even though it is of human origin (Eigenmann et al., 2013; Weksler et al., 2013; Behrens et al., 2015).

*In vitro* BBB model from primary porcine brain endothelial cells (PBECs) has been reported to show well-developed tight junctions, polarized expression of functional transporters (Patabendige and Abbott, 2014), which features comparable to that of human BBB. Additionally, the larger size of porcine brain compared to rodent brain enables higher cell yield, and it is relatively cheaper and more convenient to set up as porcine brains are by-product of the meat industry, and therefore do not require ethical approval (Patabendige et al., 2013b; Thomsen et al., 2015). On the other hand, *in silico* modeling also allows for prediction of BBB permeation of compounds particularly for passive diffusion (Abbott, 2004). Modeling based on absorption, distribution, metabolism, excretion, and toxicity (ADMET)-related descriptors predicts the effectiveness and bioavailability of compounds based on pharmacokinetic properties (Ponnan et al., 2013). Docking studies predict interaction between the compounds to their targets protein (Kitchen et al., 2004) which is also very crucial in drug designing.

Alzheimer's disease (AD) is a progressive neurodegenerative disorder which is characterized by loss of memory and other cognitive functions (Huang and Mucke, 2012). So far the US Food and Drug Administration (FDA) approved two drug classes for AD treatments which are known as AChE inhibitors and N-methyl-D-aspartate (NMDA) receptor antagonist (Deng et al., 2017). Both classes of drugs can only provide temporary and incomplete symptomatic relief accompanied with undesired side effects (Du et al., 2018). Besides that, the partial effectiveness of current AD treatments were unable to slow, reverse or thwart the progression of AD (Bhuvanendran et al., 2018; Du et al., 2018). Thus, search on the potential drugs for more effective AD treatment is urgently needed. One such promising compound is embelin (2,5- dihydroxy-3-undecyl-1,4-benzoquinone), a class of benzoquinone naturally found in the bright orange fruits of *Embelia ribes* Burm (Family: Myrsinaceae) (Kundap et al., 2017). According to Mahendran et al. (2011), embelin has been reported to show anti-inflammatory, antioxidant, analgesic, antifertility, antitumor, wound healing, hepatoprotective, and antibacterial activities. Recent reports indicated that embelin alleviates scopolamine-induced amnesia in rats and reversed memory impairment caused by streptozotocin (STZ) (Arora and Deshmukh, 2017; Bhuvanendran et al., 2018). However, the BBB permeability of embelin and its mechanism of action are unknown. Here, assessment of embelin cytotoxicity, its effect on the BBB tight junction function and BBB permeability were performed using *in vitro* PBEC BBB model; its mechanism of action was determined using AChE inhibitory assay and docking studies, to investigate its potential as a new candidate for CNS therapeutic molecule particularly for the treatment of AD.

## Materials and Methods

### Materials

Iscove's modified Dulbecco's medium (IMDM 1X), Dulbecco's modified Eagle's medium (DMEM) without Phenol Red, Hank's Balanced Salt Solution (HBSS) without calcium (Ca^2+^) and magnesium (Mg^2+^) and heat-inactivated fetal bovine serum (FBS) were purchased from Gibco Life Technologies (Grand Island, USA). Phosphodiesterase inhibitor (RO-20-1724) was obtained from Merck Chemicals Ltd. (Nottingham, UK). Corning Transwell® translucent polycarbonate filter inserts (product no. 3401, 12 mm diameter, 0.4 μm pore size, 1 × 10^8^ pores/cm^2^, 1.12 cm^2^ growth area) were obtained from Corning (New York, USA). All other chemicals were obtained from Sigma-Aldrich (Dorset, UK) unless otherwise stated.

### Isolation of Porcine Brain Microvessels and Culture of the PBECs

The porcine brain microvessels were isolated using published method (Patabendige et al., 2013a,b) with slight modifications. Porcine brains from Department of Veterinary Services Penang abattoir (Sungai Pinang, Penang, Malaysia) were transported to the lab in ice-cold IMDM supplemented with FBS (10% v/v), penicillin (100 U/mL) and streptomycin (100 μg/mL) on ice. The brains were stored at 4°C overnight prior to the isolation of microvessels due to schedule of animal slaughter at the abattoir. Microvessels obtained were stored in liquid nitrogen until further use. Here, the cryopreserved microvessels were thawed and cultured in flasks according to previous studies (Patabendige et al., 2013b) to obtain the PBECs. The PBECs were then passaged onto plates or Transwell® inserts after 4 days in culture. For cytotoxicity assay, the PBECs were cultured in 96-well plates at a seeding density of 3.2 × 10^4^ cells/well, while for TEER measurement and permeability assay, the PBECs were cultured on the Transwell® inserts at a density of 1 × 10^5^ cells/insert.

When culturing in wells and inserts, culture medium used was DMEM (with Phenol Red; Sigma D5546) supplemented with 10% (v/v) FBS, penicillin (100 U/mL), streptomycin (100 μg/mL), L-glutamine (2 mM) and heparin (125 μg/mL). To further induce BBB differentiation of the PBECs cultured on the inserts, at confluency, the culture medium was replaced by serum-free medium with added hydrocortisone (550 nM) (Hoheisel et al., 1998; Franke et al., 1999). The PBECs were also treated with 8-(4-chlorophenylthio-cAMP) (250 μM) and phosphodiesterase inhibitor (RO-20-7024) (17.5 μM) to increase tight junction tightness (Rubin et al., 1991). Cell culture was conducted at 37°C in a humidified atmosphere with 5% CO_2_ in air.

### Cytotoxicity of Embelin Toward the PBECs

MTT assay was conducted as described by Mosmann (1983) with slight modifications. Confluent PBECs in 96-well plate were incubated with embelin prepared in the culture medium at concentrations ranging from 10 to 100 μg/mL, for 1 h at 37°C. After 1 h, embelin solution was discarded and the PBECs were incubated with 100 μL MTT solution (1 mg/mL) prepared in DMEM without Phenol Red for 4 h at 37°C. Untreated cells were used as control to represent total viable cells. The cells were also treated with 1% (v/v) methanol in culture medium as vehicle control. After 4 h, the MTT solution was removed and replaced with 100 μL of propan-2-ol to dissolve formazan crystals formed. Absorbance was measured at 560 nm and 690 nm using Multiskan Go Microplate Reader (Thermo Fisher Scientific, MA, USA). The experiment was conducted in triplicate, in three independent experiments. Percentage of cell viability was calculated using following equation:

(1) $$\begin{array}{l} \%\;of\;cell\;viability\\ =\;\frac{(Absorbance_{560}-Absorbance_{690})\;of\;treated\;cells}{(Absorbance_{560}-\;Absorbance_{690})\;of\;untreated\;cells}\;\times 100 \end{array}$$

### Real-Time TEER Assay

The assay was conducted to assess effect of embelin on the PBEC tight junction function. Approximately 24 h after the serum-free medium change and treatment with 8-CPT-cAMP and RO-20-1724, TEER of the PBEC monolayer was measured using WPI STX-100C chopstick electrode pair connected to EVOM meter (World Precision Instruments Inc., Sarasota, FL, USA) for 30 min at 1 min interval. After minute 10 TEER was recorded, embelin (30 μg/mL), DMEM (negative control), 100% DMSO (positive control) and 1% (v/v) methanol in DMEM were added to inserts separately and the TEER measurement was resumed until minute 30. The inserts were returned to the incubator for 30 min, then taken out again to measure TEER at minute 60. DMEM and 100% DMSO were used as negative and positive controls respectively for causing a leaky tight junction while 1% (v/v) methanol was used as vehicle control as the embelin solution contained methanol. TEER values of the cell monolayer were subtracted from value recorded for blank insert (without cells) and multiplied by growth surface area as shown by the following equation:

(2) $$TEER\;(\Omega .cm^{2})=\left(R_{cell\;monolayer}-\;R_{blank}\right)\times A$$

in which, R_cell monolayer_ is the resistance (Ω) of insert with cells, R_blank_ is the resistance (Ω) of blank insert without cells and A is the surface area of insert (1.12 cm^2^). For each insert, the TEER values obtained at the different time points were then normalized to initial measurement at t = 0 min, and results are reported as percentage of initial TEER. Mean TEER of the PBEC monolayer used for the investigated groups before the starts of the assay are: 427.60 ± 109.39 Ω.cm^2^ for embelin; 427.00 ± 71.45 Ω.cm^2^ for DMEM; 456.40 ± 87.28 Ω.cm^2^ for 100% DMSO; and 423.40 ± 52.53 Ω.cm^2^ for 1% (v/v) methanol.

### *In vitro* BBB Permeability Assay

Quality control value for cell monolayer TEER was set at 200 Ω.cm^2^. Here, average cell monolayer TEER obtained was 365.37 ± 113.00 Ω.cm^2^, therefore deemed suitable to be used for the permeability assay. Briefly, DMEM without Phenol Red supplemented with HEPES (25 mM) at pH 7.4 was used as buffer. Embelin was dissolved in methanol at 3 mg/mL and diluted in the buffer to obtain a concentration of 30 μg/mL. NaF, a paracellular permeability marker compound was added to the embelin solution at concentration of 5 μM. To start the assay, the culture medium in the apical (filter insert) and the basolateral (well) compartments was aspirated and the filter inserts containing the PBECs were transferred to a 12-well plate containing the pre-warmed buffer (1,500 μL) on a shaker-incubator (THERMOstar, BMG Labtech, Germany). To start the experiment, 500 μL of the embelin solution was added to the apical compartment. The assay was carried out at 37°C for 60 min under stirring condition at 150 rpm. At the end of the assay, samples were taken from each compartment (400 μL from the apical and 1,200 μL from the basolateral) for analysis.

The samples were processed using liquid-liquid extraction method using chloroform (organic phase) to extract embelin from the buffer (aqueous phase), followed by drying using nitrogen gas. When dried, methanol was added to tubes to re-dissolve embelin and the samples were analyzed using liquid chromatography tandem mass spectrophotometry (LC-MS/MS). Fluorescence of NaF was measured at 485 nm excitation and 535 nm emission using a fluorescence intensity plate reader (CHAMELEON™ V, Hidex, Finland). Apparent permeability (*P*_app_, cm/s) of embelin and NaF was calculated using the following equation:

(3) $$P_{app}(x\;10^{-6}cm/s)=\;\frac{C_{R}.\;V_{R\;}}{C_{D}.V_{D}.t.A\;}\;V_{D}$$

in which C_R_ and C_D_ are embelin concentrations (mol/cm^3^) in the receiver and donor compartments i.e., basolateral and apical compartment respectively, V_R_ and V_D_ are the volumes in the receiver compartment (1,500 μL) and the donor compartment (500 μL), t is the incubation time (60 min), and A is the surface area of the filter insert (1.12 cm^2^). Values obtained were divided by 60 to express results in cm/s.

Percentage of recovery of the compounds was calculated using the following equation:

(4) $$\%\;of\;recovery={\;}_{\;}^{\;}\;\frac{AmtD_{t=60}+AmtR_{t=60}}{AmtD_{t=0}}\;X\;100\%$$

in which AmtD_t = 60_ and AmtR_t = 60_ are amount of compound in the donor and the receiver compartments i.e., apical and basolateral compartment respectively at 60 min, and AmtD_t = 0_ is amount of compound in the donor compartment at initial (*t* = 0 min).

### LC-MS/MS for Quantification of Embelin

The concentrations of embelin in the apical and basolateral compartments from the BBB permeability assay were quantified using LC-MS/MS. Standard solutions of embelin were prepared in methanol with concentrations of 1, 2, 5, 7.5, and 10 μg/mL. The standard solutions and samples from the assay were injected at 10 μL into Agilent 6410 Triple Quad LC/MS comprising ZORBAX Eclipse plus C18 RRHD 2.1 × 150 mm and 1.8 μm column at a flow rate of 0.5 mL/min. The mobile phase was consisted of 0.1% formic acid in water (solvent A) and 0.1% formic acid in acetonitrile (solvent B) with a total run time of 4 min. The gradient elution was set as (i) 0–1 min, 75% B; (ii) 1–2 min, 90% B; (iii) 2–3 min, 95% B; (iv) 3–4 min, 100% B. Electrospray ionization mass spectrometry condition was programmed with gas temperature of 300°C, nebulizer pressure of 40 psi, capillary voltage of 4,000 V and drying gas flow at 10.0 L/minute. The MS scan parameters had a dwell time of 250 s with two products of 122.9 and 96 Da, performed in negative polarity mode.

### *In vitro* AChE Inhibitory Assay

AChE inhibition of embelin was evaluated using the Ellman's method (Ellman et al., 1961; Liew et al., 2015) with slight modifications. A serial dilution of embelin which highest concentration lesser than 200 μM was prepared using DMSO and 0.1 M sodium phosphate buffer (pH 7.8), with DMSO final concentration of <1% (v/v). Sodium phosphate buffer (140 μL) was added to 96-well plate followed by sample solution (20 μL) and absorbance was measured at 412 nm. This reading served as blank. Then, AChE enzyme from electric eel (0.2 U/mL, 20 μL) was added to the wells and incubated for 15 min at room temperature. Finally, 5,5′-dithiobis (2-nitrobenzoic acid) (DTNB) (3 mM, 10 μL) was added, followed by addition of acetylthiocholine iodide (ATCI) (15 mM, 10 μL). The rate of absorbance change was measured at 412 nm for 30 min with a Multiskan Go Microplate Reader (Thermo Fisher Scientific, MA, USA). Each assay was carried out with donepezil as positive control (0.015 μM). The reactions were performed in triplicates, in three independent experiments and the IC_50_ values were determined from inhibition vs. concentration plot. Below is the equation to calculate AChE inhibition.

(5) $$Percentage\;inhibition\;\left(\%\right)=\left[\;1-\left(\frac{Sample}{Control}\right)\right]\times 100$$

### Molecular Docking

All molecular docking studies were performed on Biovia Discovery Studio (BDS) 4.5 (www.3dsbiovia.com). For AChE, the x-ray crystal structure of AChE complexed with anti-Alzheimer drug (donepezil or E2020) was retrieved from the Protein Data Bank (PDB code: 1EVE) (Kryger et al., 1999). The water molecules were deleted and hydrogen atoms were added. Finally protein was refined with CHARMm at physiological pH. To validate the docking reliability, co-crystalized ligand donepezil was first re-docked to the binding site of AChE. Consequently, embelin was docked into same active site; 30 conformations of the compound were obtained through CDOCKER. The conformations with lowest energy were selected as the most probable binding conformation for each ligand. Docking studies was further carried out with Aβ peptide. Four receptors were chosen for Aβ peptide docking including monomers Aβ _1−40_, Aβ _1−42_ and fibril fragments 6Aβ _9−40_ and 5Aβ _17−42_ (Petkova et al., 2002, 2006; Lührs et al., 2005; Ngo and Li, 2013). The structures of Aβ were retrieved from Protein Data Bank and respective PDB ID are shown in **Table 3**. Embelin was docked by using CDOCKER program. The BBB prediction for embelin was also calculated using BDS 4.5.

### Statistical Analysis

Statistical analyses were performed using GraphPad Prism 5.0 software (La Jolla, CA). All data are presented as mean ± SD and the samples were analyzed using One-way ANOVA and unpaired *t*-test. Statistical significance was reported as follows: ^*^P < 0.05, ^**^P < 0.01, ^***^P < 0.001.

## Results

### Cytotoxicity of Embelin Toward the PBECs

**Figure 1:**
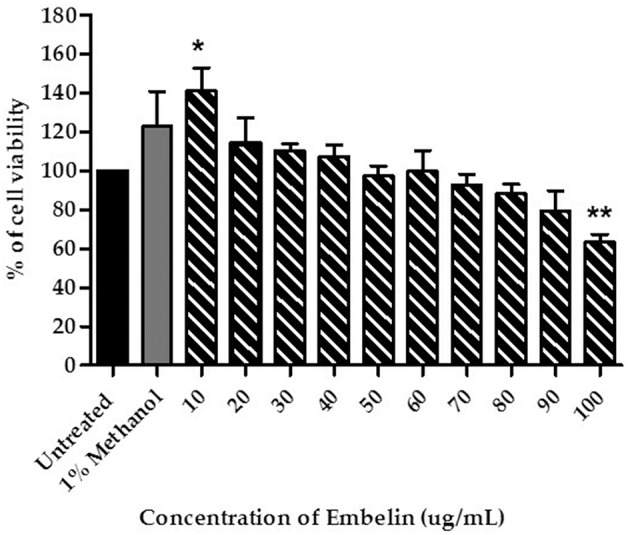
Cytotoxicity of embelin toward primary porcine brain endothelial cells (PBECs), tested using the MTT assay. Untreated cells were used as a control to represent total viable cells. The cells were also treated with 1% (v/v) methanol in culture medium as vehicle control. Data are mean ± SD from 3 independent experiments (n = 3) with 3 replicates for each experiment. * *P* < 0.05 ** *P* < 0.01, as tested using One-way ANOVA.

Prior to the BBB permeability assay, viability of the PBECs in presence of embelin was determined. One-way ANOVA analysis shows a significant difference between the treatment groups and the cell viability (*F* = 6.134; *P* < 0.001). As shown in Figure 1, the PBECs treated with embelin from 10 to 90 μg/mL did not show reduction in viability when compared to the untreated cells. However, embelin at 100 μg/mL caused reduction in PBEC viability (*P* < 0.01) compared to the untreated cells. Interestingly, embelin tested at 10 μg/mL caused an increase in cell viability (*P* < 0.05).

### Real-Time TEER Assay

**Figure 2:**
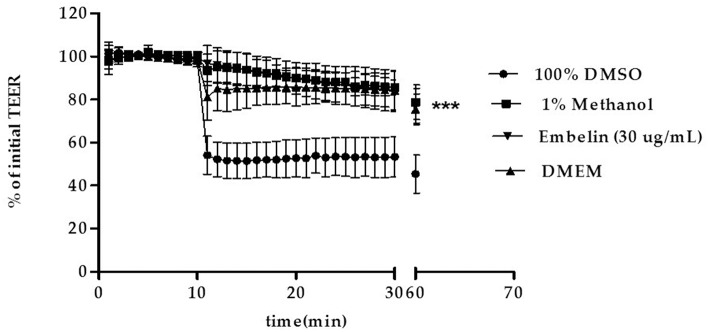
Effect of embelin on PBEC barrier function. Transendothelial electrical resistance (TEER) across the PBEC monolayer was measured for 30 min at 1 min interval using WPI STX-100C chopstick electrode pair connected to EVOM meter, followed by TEER measurement at minute 60. Embelin (30 μg/mL), DMEM (negative control), DMSO (100%; positive control) and methanol (1% v/v; vehicle control) were added separately to the inserts after minute 10 TEER was recorded. Data are presented as mean ± SD, *n* = 3 independent experiments. *** *P* < 0.001, as tested using One-way ANOVA.

Tight junctions integrity of the PBEC monolayer was determined by measuring the TEER at 1 min interval up to 30 min, then the cells returned to the incubator, followed by measurement of TEER at minute 60. Embelin was tested at 30 μg/mL, Dulbecco's modified Eagle's medium (DMEM) and dimethyl sulfoxide **(**DMSO) were used as negative and positive control respectively. As shown in Figure 2, embelin at 30 μg/mL did not disrupt the tight junction integrity during the 1 h exposure and it was significant at *P* < 0.001 when compared to 100% DMSO.

### *In vitro* BBB Permeability Assay

Permeability assay is conducted to measure the rate of BBB crossing for compounds. In this study, the rate at which embelin transverse across the PBEC monolayer from apical-to-basolateral, blood-to-brain side was measured and reported as apparent permeability (*P*_app_, cm/s). As shown in Table 1, embelin demonstrated *P*_app_ value of 35.46 ± 20.33 × 10^−6^ cm/s with 83.53% recovery. Sodium fluorescein (NaF) as paracellular permeability marker compound showed low *P*_app_ of 2.47 ± 1.63 × 10^−6^ cm/s, indicating that the tight junctional integrity was preserved during the assay.

**Table 1:** *P*_app_ values and % recovery of embelin and NaF.

**Compound**	***P*_**app**_ (x 10^**−6**^cm/s)**	**% Recovery**
Embelin	35.46 ± 20.33	83.53 ± 14.72
NaF	2.47 ± 1.63**	78.16 ± 3.63

** *P < 0.01 when compared to embelin using unpaired t-test*.

### AChE Inhibitory Assay

**Figure 3:**
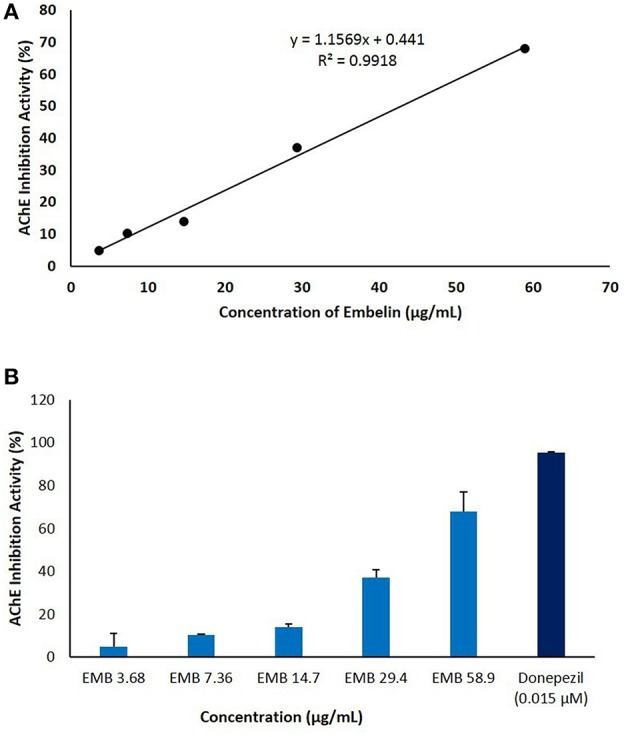
The anti-cholinesterase activity of embelin (3.68–58.9 μg/mL) using *in vitro* AChE inhibitory assay. The graph was plotted by keeping embelin concentration on X-axis against AChE inhibition activity (%) on Y-axis. **(A)** IC_50_ value was calculated using standard curve generated using Microsoft Excel. **(B)** AChE inhibitory activity (%) of embelin compared to donepezil.

Embelin was evaluated for its inhibitory activity of AChE from electric eel (*Electrophorus electricus*). Donepezil was used as a positive control and to validate the assay by comparing IC_50_ value obtained in this study with reported values (Liew et al., 2015). Embelin was tested at a series of concentration from 3.68 to 58.9 μg/mL in order to determine the IC_50_ value using standard curve generated using Microsoft Excel. As shown in Figure 3, IC_50_ value obtained for embelin against AChE is 49.61 μg/mL.

### Molecular Docking

The results for docking studies are expressed as interaction energy in -kcal/mol. The docked conformations of donepezil and embelin and key interactions are summarized in Table 2. Based on the results, embelin has better binding to the AChE active site with the interaction energy of −65.75 kcal/mol compared to E2020. Likewise, the docked conformations of embelin and Aβ and key interactions are summarized in Table 3. Binding to fibril 6Aβ _9−40_ and 5Aβ _17−42_ display high interaction energy of −54.01 and −38.77, respectively when compared with Aβ monomers.

**Table 2:** Binding modes of embelin docked to AChE active sites.

**Compounds**	**Docked pose**	**CDocker interaction energy** **(-kcal/mol)**	**Non-bond interactions**
E2020 (reference)	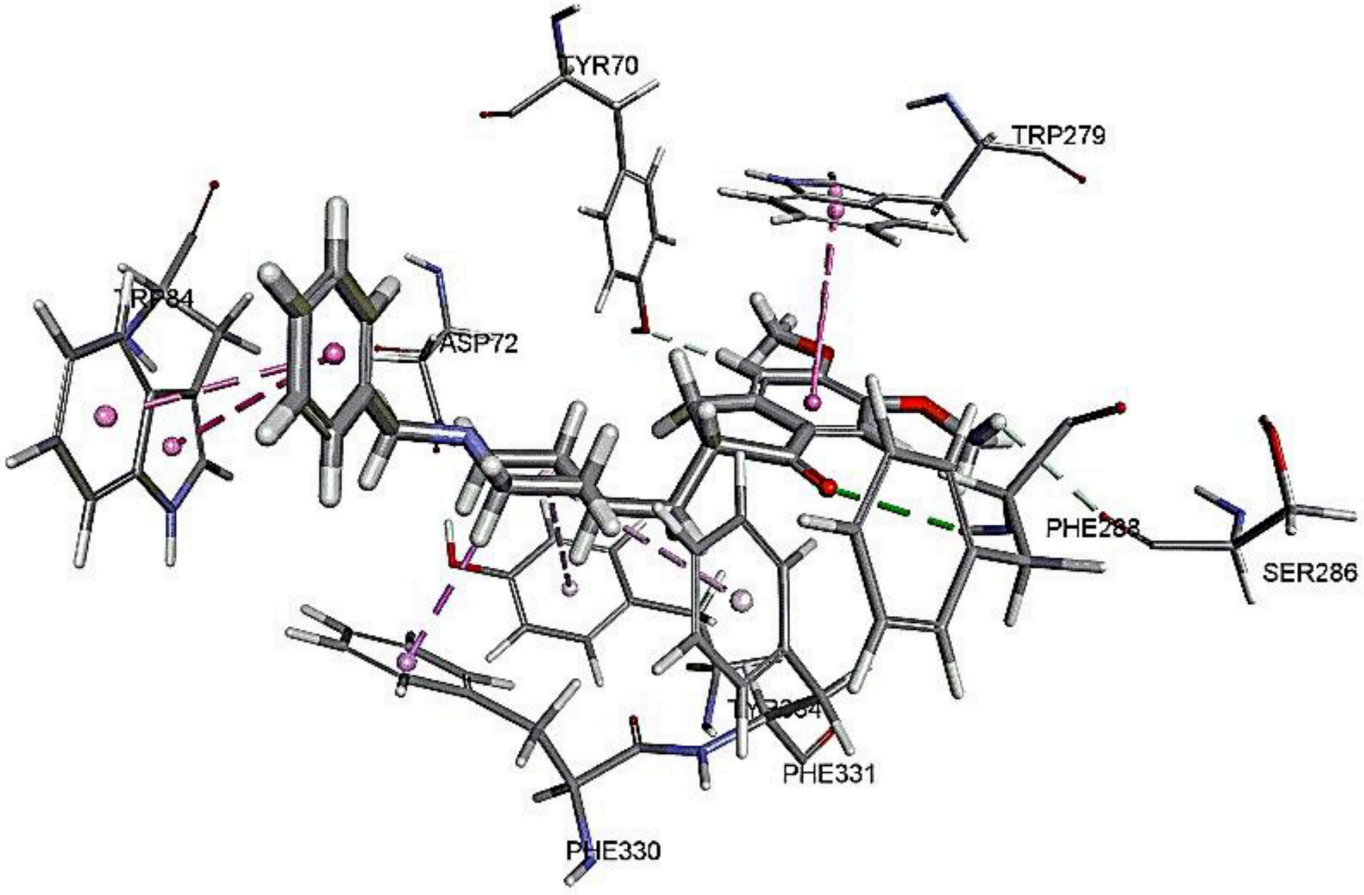	48.5319	Hydrogen Bonds E2020 to PHE288 E2020 to ASP72 E2020 to SER286 E2020 to TYR70 Hydrophobic interactions -Pi-Sigma E2020 to PHE330 E2020 to TRP84 E2020 to TRP279 E2020 to PHE331 E2020 to TYR334
Embelin	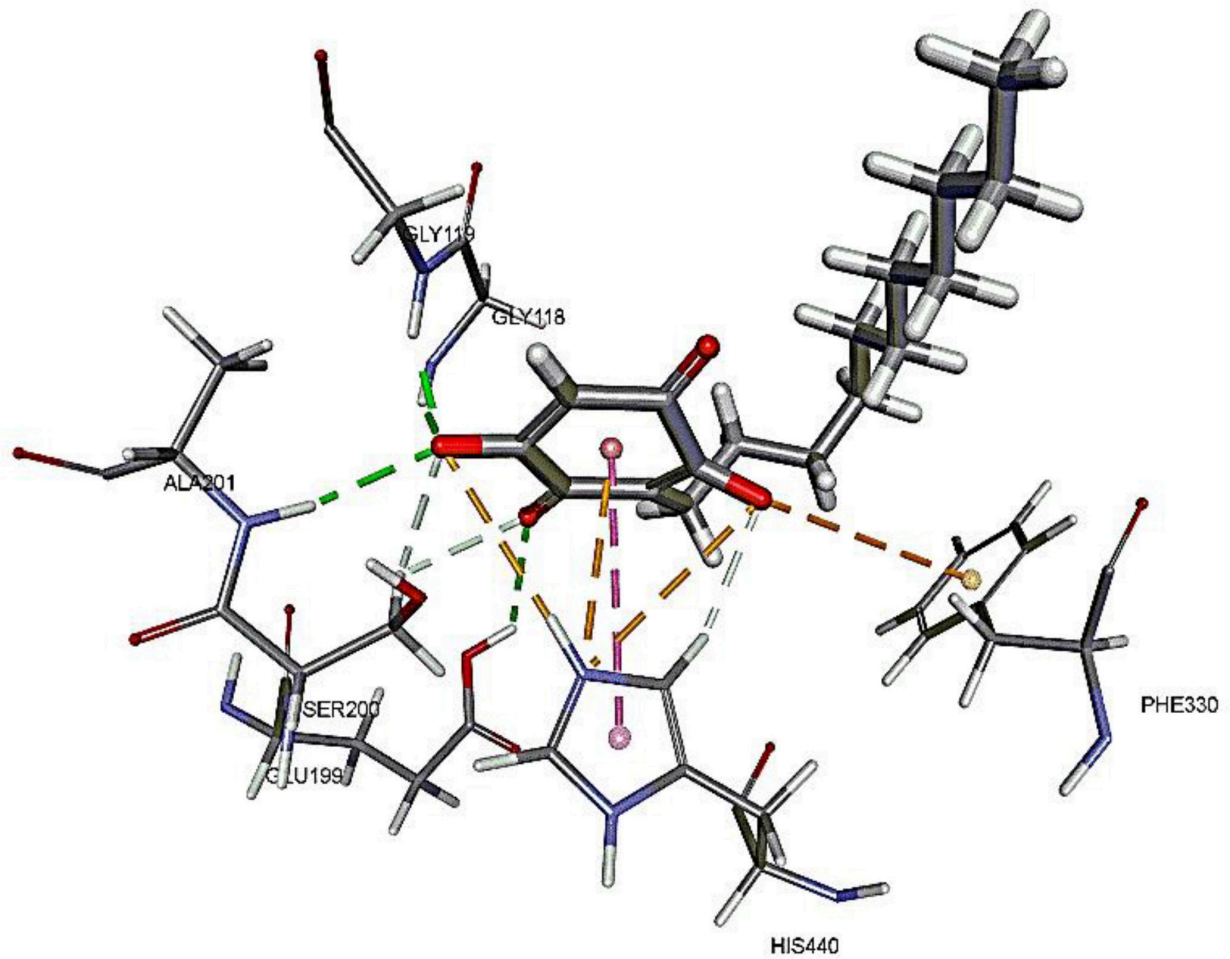	65.7525	Hydrogen Bonds Embelin to GLY118 Embelin to GLY119 Embelin to GLY199 Embelin to ALA201 Embelin to SER200 Embelin to HIS440 Hydrophobic interactions -Pi-Pi Embelin to HIS440 Electrostatic interactions HIS440 to Embelin Embelin to PHE330

**Table 3:** Binding modes of embelin docked to Aβ active sites (monomers and fibrils).

**PDB ID**	**Docked pose**	**CDocker interaction energy (-kcal/mol)**	**Non-bond interactions**
1BA4 (Aβ _1−40_)	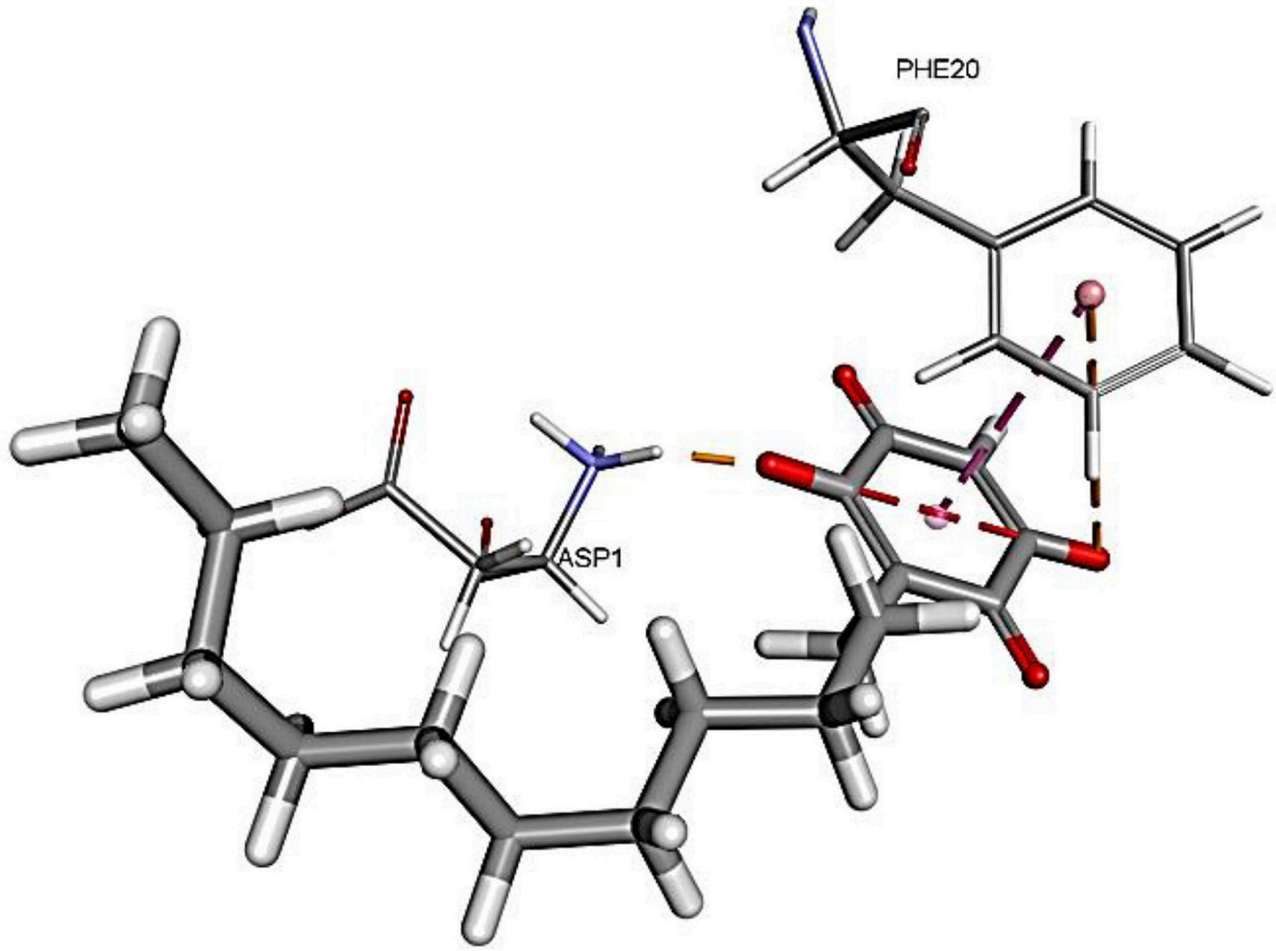	34.1594	Hydrogen Bonds Embelin to ASP1 Hydrophobic interactions Embelin to PHE20 Electrostatic interactions Embelin to PHE20
1Z0Q (Aβ _1−42_)	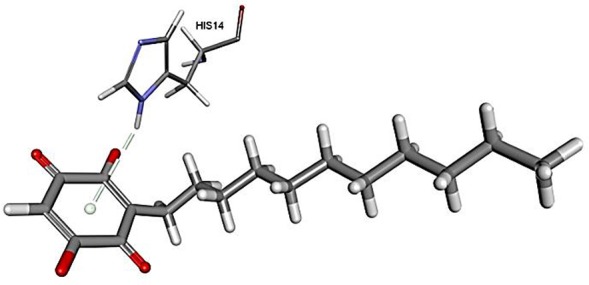	24.2574	Hydrogen Bonds Embelin to HIS14
2BEG (5Aβ _17−42_)	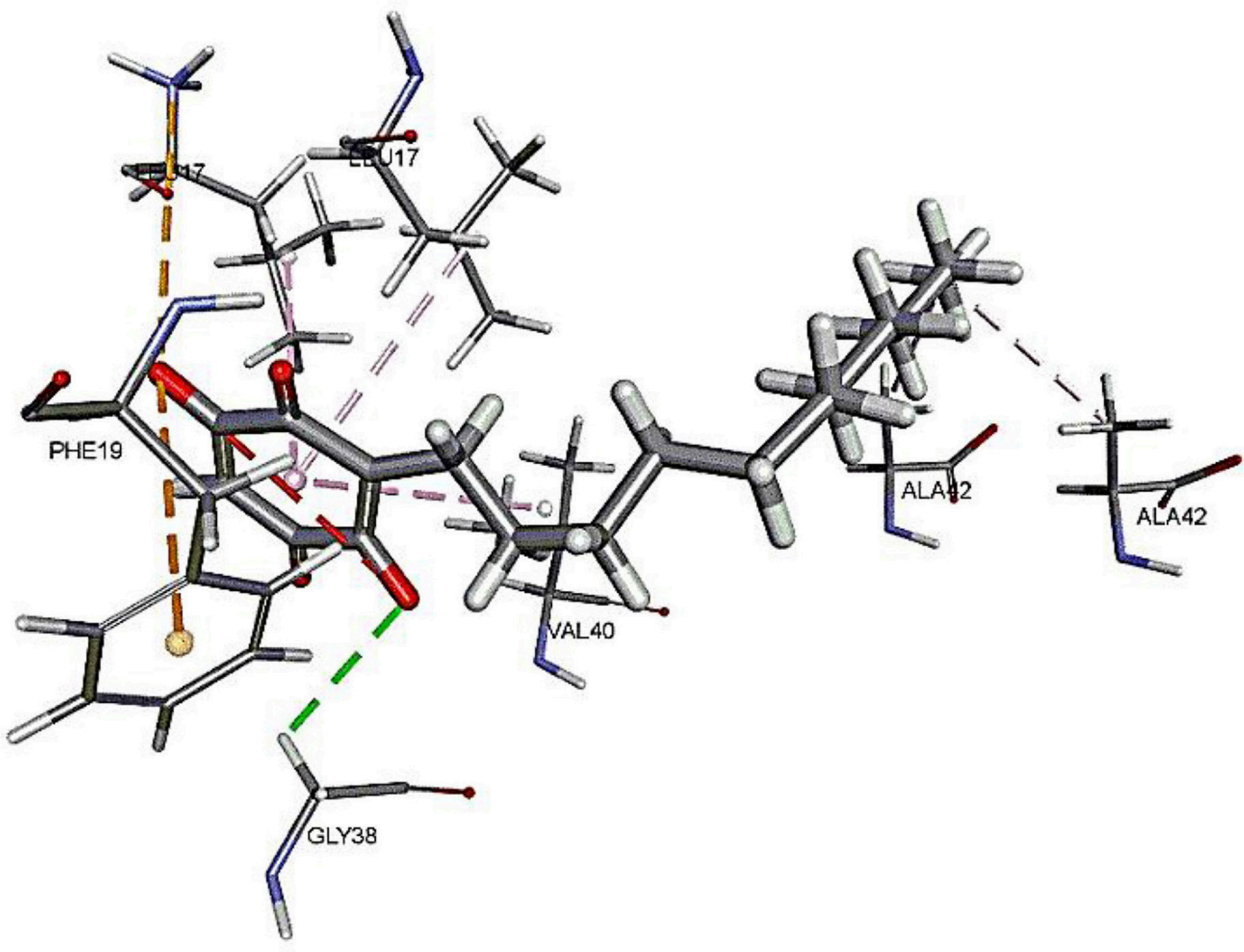	38.7666	Hydrogen Bonds Embelin to GLY38 Hydrophobic interactions Embelin to ALA42 Embelin to ALA42 Embelin to LEU17 Embelin to LEU17 Embelin to VAL40 Electrostatic interactions Embelin to PHE19
2LMN (6Aβ _9−40_)	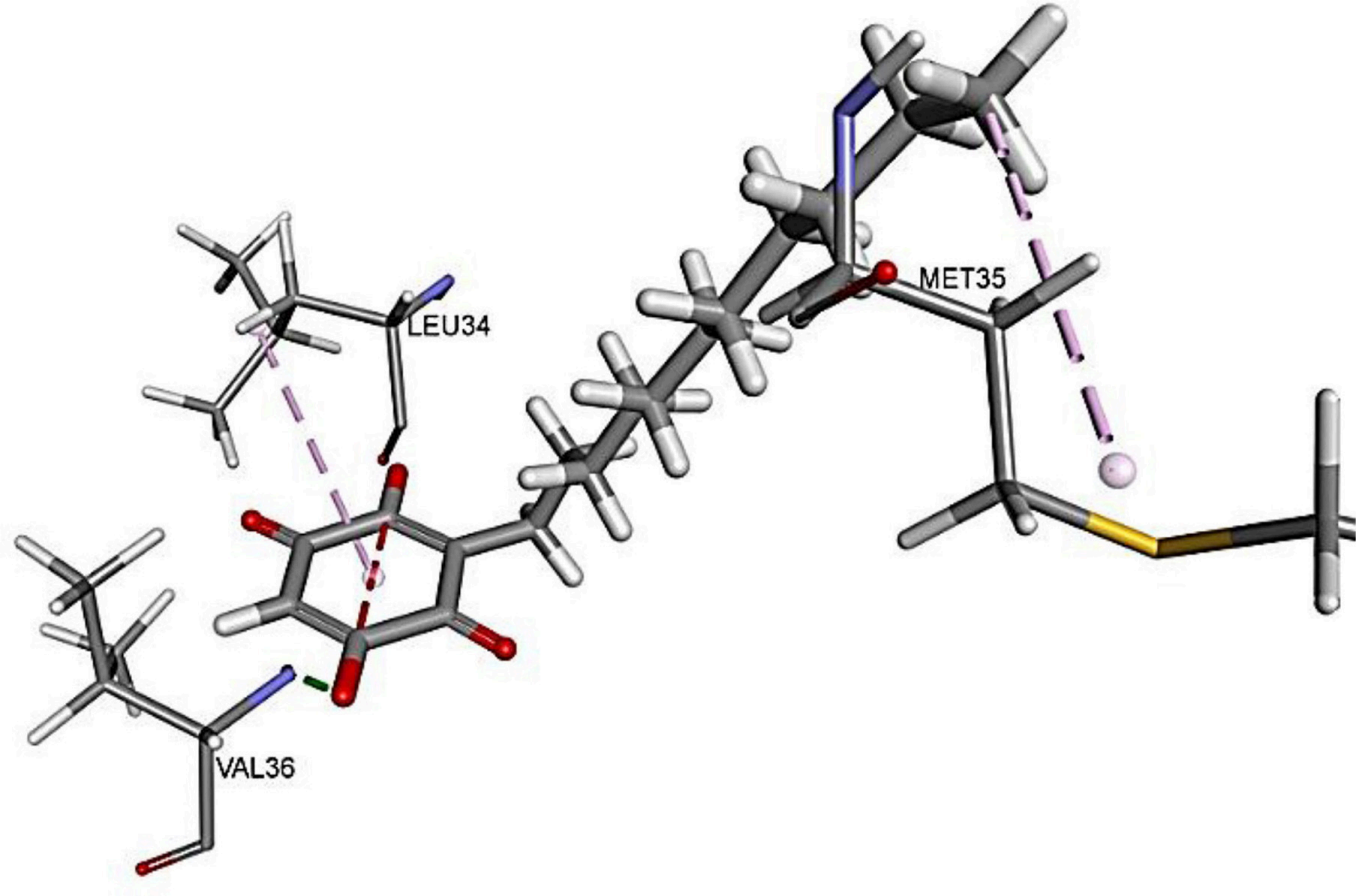	54.0122	Hydrogen Bond Embelin to VAL36 Hydrophobic interactions Embelin to MET35 Embelin to LEU34

## Discussion

To date, not a single study reported on BBB permeability of embelin (Kundap et al., 2017). According to Pathan et al. (2009) in order to cross the BBB, a compound should be in unionized form, molecular weight of <400 Da, log *P*-value near to 2 and around 8–10 hydrogen bonds (Pathan et al., 2009). Embelin has all these characteristics, hence high possibility to permeate the BBB. Cell culture models are the most favored tools for assessing BBB permeation of compounds, giving information on passive permeability across cell membranes and also on carrier-mediated transport (Hakkarainen et al., 2010). Therefore, we conducted *in vitro* BBB permeability assay of embelin using PBEC BBB model. Prior to the permeability assay, we established that embelin does not cause any toxicity to the PBECs up to 90 μg/mL, and embelin at 30 μg/mL does not affect BBB tight junctional integrity compared to the 100% DMSO during the 1 h exposure. For the permeability assay, cell monolayer with TEER values exceeding 200 Ω.cm^2^ was used as the cells were considered to have minimal tight junction leakiness (Gaillard and de Boer, 2000). From the results, embelin demonstrated high *P*_app_ value which is comparable to that of donepezil reported by Liew et al. (2017).

The high *P*_app_ value of embelin could consists of one or a combination of routes used by the compound to cross the BBB. Embelin could permeate via passive transcellular route across the cell membrane only or at the same time facilitated by membrane transporter expressed on cell membranes. To further dissect the mechanisms involved, bidirectional permeability assay could be conducted (Liew et al., 2017). Higher apical-to-basolateral (blood-to-brain side) permeability compared to basolateral-to-apical permeability indicates facilitative transport by influx transporter, net permeability in the opposite direction indicates efflux.

Additionally, *P*_app_ value of embelin is much higher than *P*_app_ of the paracellular marker compound used in this study i.e., NaF (Table 1). This could indicate that embelin largely cross the BBB via transcellular route and not paracellular route (via the tight junction) *in vitro*. This is further supported by the outcome of ADMET for BBB penetration for embelin which is level 1. According to Ponnan et al. (2013), ADMET BBB penetration level 1 indicates high penetration of a compound across the BBB after an oral administration.

The CDOCKER was used for docking of all compounds. The CDOCKER is CHARMm-based docking algorithm that uses the CHARMm family of force fields and offers all the advantages of full ligand flexibility (including bonds, angles, and dihedrals) and reasonable computation times (Brooks et al., 1983). The CDOCKER algorithm adopts a strategy involving the generation of several initial ligand orientations in the active site of target protein followed by molecular dynamics-based simulated annealing and final refinement by energy (Mo et al., 2012).

The molecular docking study was carried out to understand the binding mode of embelin within the active site of AChE using Discovery Studio suit 4.5 software. The x-ray crystal structure of AChE complexed with donepezil (or E2020) was retrieved from Protein Data Bank (PDB code: 1EVE). To validate the docking protocol, donepezil was first docked into AChE active site. As revealed by Kryger et al. (1999) phenyl ring of E2020 formed π-stacking with Trp 84 and Phe 330 while another aromatic ring stacked with Trp279. Further, hydrogen bond was observed between Phe288 and ketone oxygen. The root mean square deviation (RMSD) and CDOCKER interaction energy (CDIE) were found to be 1.28 Å and −48.53 kcal/mol, respectively (Kryger et al., 1999). Embelin showed a promising favorable interaction with AChE binding site with CDOCKER interaction energy of −65.75 kcal/mol. This finding is consistent with AChE inhibitory activity of embelin. Higher binding interaction energy indicating embelin may bound to the AChE active site which likely to trigger the catalytic site for its inhibitory activity for AChE (Liew et al., 2015).

Accumulation of research evidence over the last 20 years revealed that Aβ oligomers is associated with AD pathogenesis (Hayden and Teplow, 2013). Therefore, there is a pressing need to find compounds that are able to promote anti-Aβ aggregation and clearance (Ngo and Li, 2013). There are studies reported the potential of small molecules in converting toxic oligomers into non-toxic amorphous aggregates (Ehrnhoefer et al., 2008; Bieschke et al., 2010). Furthermore, small molecules could also contribute in morphological changes of amyloid fibrils to inert form (Dzwolak et al., 2005; Sibley et al., 2008). Since Aβ peptides are located in the brain, an efficient drug should be able to cross the BBB to interfere with their activities (Ngo and Li, 2013). Similar to AChE docking study, embelin also interacted favorably with Aβ peptides as evident from CDOCKER interaction energy as shown in Table 3. These results revealed that embelin has potential to bind with Aβ peptides which may then slow down or degrade mature fibrils of Aβ peptides.

## Conclusion

This study for the first time has demonstrated the use of *in vitro* PBEC BBB model in the evaluation of embelin BBB permeability. This cell-based model showed that embelin is able to cross the BBB which further supported by *in silico* results. Besides that, this study has found embelin as a promising AChE inhibitor as evident from the AChE inhibition assay. Using molecular docking, we could predict that embelin has favorable binding mode within the AChE and Aβ peptide active sites. Hence, based from this study we discovered that embelin is a favorable compound which can be further developed into a potential therapeutic multipotent agent for AD.

## Author Contributions

SB conceived, performed experiments and wrote the manuscript. NH helped in PBEC *in vitro* studies and gave valuable input in writing the paper. NA performed molecular docking and helped in writing of manuscript. IO was involved in LC-MS/MS and gave critical feedback for this study. SY and MS were involved in conceptualization, designing the study, interpreted, supervised the study and contributed in writing of the manuscript. All the authors read and approved the final manuscript.

### Conflict of Interest Statement

The authors declare that the research was conducted in the absence of any commercial or financial relationships that could be construed as a potential conflict of interest.

## Abbreviations

ADMET: Absorption, distribution, metabolism, excretion and toxicity
AChE: Acetylcholinesterase
Aβ: Amyloid beta
ATCI: Acetylthiocholine iodide
BBB: Blood-brain barrier
CNS: Central nervous system
CDIE: CDOCKER interaction energy
DMEM: Dulbecco's modified Eagle's medium
DMSO: Dimethyl sulfoxide
DTNB: 5,5′-dithiobis (2-nitrobenzoic acid)
FBS: Fetal bovine serum
HBSS: Hank's Balanced Salt Solution
hCMEC/D3: Human cerebral microvascular endothelial cell line
IMDM: Iscove's modified Dulbecco's medium
LC-MS/MS: Liquid chromatography tandem mass spectrophotometry
MTT: 3-(4, 5-dimethylthiazol-2-yl)-2, 5-diphenyltetrazolium bromide
NaF: Sodium fluorescein
*P*_app_: Apparent permeability
PBECs: Primary porcine brain endothelial cells
PDB: Protein Data Bank
RMSD: Root mean square deviation
STZ: Streptozotocin
TEER: Transendothelial electrical resistance.
